# Accuracy and Tuning of Flow Parsing for Visual Perception of Object Motion During Self-Motion

**DOI:** 10.1177/2041669517708206

**Published:** 2017-05-18

**Authors:** Diederick C. Niehorster, Li Li

**Affiliations:** Department of Psychology, The University of Hong Kong, Pokfulam, Hong Kong; Department of Psychology, The University of Hong Kong, Pokfulam, Hong Kong; Neural Science Program, NYU-ECNU Institute of Brain and Cognitive Science, New York University Shanghai, China

**Keywords:** self-motion, optic flow, flow parsing, global motion, speed tuning

## Abstract

How do we perceive object motion during self-motion using visual information alone? Previous studies have reported that the visual system can use optic flow to identify and globally subtract the retinal motion component resulting from self-motion to recover scene-relative object motion, a process called flow parsing. In this article, we developed a retinal motion nulling method to directly measure and quantify the magnitude of flow parsing (i.e., flow parsing gain) in various scenarios to examine the accuracy and tuning of flow parsing for the visual perception of object motion during self-motion. We found that flow parsing gains were below unity for all displays in all experiments; and that increasing self-motion and object motion speed did not alter flow parsing gain. We conclude that visual information alone is not sufficient for the accurate perception of scene-relative motion during self-motion. Although flow parsing performs global subtraction, its accuracy also depends on local motion information in the retinal vicinity of the moving object. Furthermore, the flow parsing gain was constant across common self-motion or object motion speeds. These results can be used to inform and validate computational models of flow parsing.

## Introduction

How do we perceive object motion during self-motion? When we are stationary and not making any head or eye movements, an object’s movement is defined directly by its retinal motion. However, when we move, the optical motion of the object is confounded by self-motion and becomes the sum of the object’s movement and optic flow, a global, complex pattern of optical motion of all elements in the world that is due to self-motion (Gibson, 1950, 1954). To accurately perceive object motion in this case, the visual system must solve the problem of distinguishing between these two components in retinal motion.

It has long ago been proposed that the coherent large pattern of optical flow, normally generated by movements of the observer, specifies how one has just moved (Gibson, 1958). Deviations from this global flow pattern signal independent object motion (Gibson, 1954). Indeed, it has been shown that the visual system is sensitive to such deviation information for the purpose of detecting object motion during self-motion (Bravo, 1998; Royden & Connors, 2010; Royden & Moore, 2012; Royden, Wolfe, & Klempen, 2001; Rushton, Bradshaw, & Warren, 2007; van Loon, Hooge, & van den Berg, 2003). To explain the underlying perceptual process for object motion perception during self-motion, Rushton and Warren (2005) proposed the *flow parsing* hypothesis. That is, the visual system uses retinal flow to determine what component of retinal motion is due to self-motion. It then globally parses out this component and leaves the observer with a percept of scene-relative object motion.

The flow parsing hypothesis is supported by the findings of a series of studies using displays that simulated self-motion (Matsumiya & Ando, 2009; Rushton & Warren, 2005; Warren & Rushton, 2007, 2008, 2009a, 2009b; Warren, Rushton, & Foulkes, 2012; see also Gray, Macuga, & Regan, 2004; Reinhardt-Rutland, 2003). Specifically, Warren and Rushton (2009b) presented observers with displays in which a probe moved over a background optic flow that simulated forward translation. They removed one hemifield of the optic flow pattern and placed the moving probe object in the empty hemifield at one of two eccentricities. The probe at the larger eccentricity was further away from the hemifield containing optic flow. Strikingly, the perceived motion trajectory of the more eccentric probe was tilted further away from its retinal trajectory, a phenomenon that is hard to explain by local motion contrast effects. However, because the global flow speed increases with eccentricity, this finding is consistent with the idea that flow parsing involves identifying and globally removing components of retinal motion resulting from self-motion.

Although previous studies have shown that the visual system performs flow parsing to recover scene-relative object motion during self-motion, it is still unknown how accurately we can perceive object motion during self-motion using visual information alone. This question arises as it is possible that nonvisual information about self-motion such as provided by vestibular and proprioceptive information and efference copies of motor commands is required for fully removing the retinal effects of self-motion. It is also unknown whether the accuracy of scene-relative object motion perception changes with self-motion or object motion speed, to what extent flow parsing can subtract out common global motion in the display, and what role local motion mechanisms play in this process (e.g., Braddick, 1993; Farrell-Whelan, Wenderoth, & Wiese, 2012; Gogel, 1979; Loomis & Nakayama, 1973; Nawrot & Sekuler, 1990; Paffen, Tadin, te Pas, Blake, & Verstraten, 2006; Post, Chi, Heckmann, & Chaderjian, 1989).

The current study aimed to examine these key properties of the flow parsing process to shed light on the neural computations underlying flow parsing. Specifically, in Experiment 1, we developed a retinal motion nulling method to measure and quantify the extent to which flow parsing removes the motion component due to self-motion from the retinal motion of a moving object (i.e., flow parsing gain) during forward self-motion. The stereo display simulated an observer moving toward a rigid cloud of objects while a probe object moved in the cloud. We varied the availability of two types of local motion information originating from different parts of three-dimensional (3D) space surrounding the probe object to examine the importance for flow parsing of local motion information originating from the same depth as the moving object, and of motion information originating from the retinal vicinity of the probe object. In Experiments 2 and 3, we further used our method to examine the tuning of flow parsing to self-motion and object motion speed. This was to characterize how the accuracy of flow parsing depends on self-motion and object motion speed. The findings of these experiments would help develop a more detailed understanding of how flow parsing makes use of various types of local motion information as well as how the process is tuned to self-motion and object motion speed and would thus inform computational and neural models of flow parsing (e.g., Layton & Fajen, 2016) as well as provide the data required for validating these models.

## Experiment 1: Flow Parsing Gain and Local Motion Information

Previous studies that found supporting evidence for flow parsing examined whether the perceived tilt of a moving object’s trajectory was consistent with the predictions of flow parsing (Warren & Rushton, 2007, 2008, 2009b; Warren et al., 2012), whether the perceived 3D object motion was in a world-centered reference frame (Matsumiya & Ando, 2009), or whether the direction of scene-relative object motion was correctly perceived (Rushton & Warren, 2005; Rushton et al., 2007; Warren & Rushton, 2009a). It still remains in question how accurate flow parsing is during forward self-motion, a commonly experienced form of self-motion in daily life.

**Figure 1.**
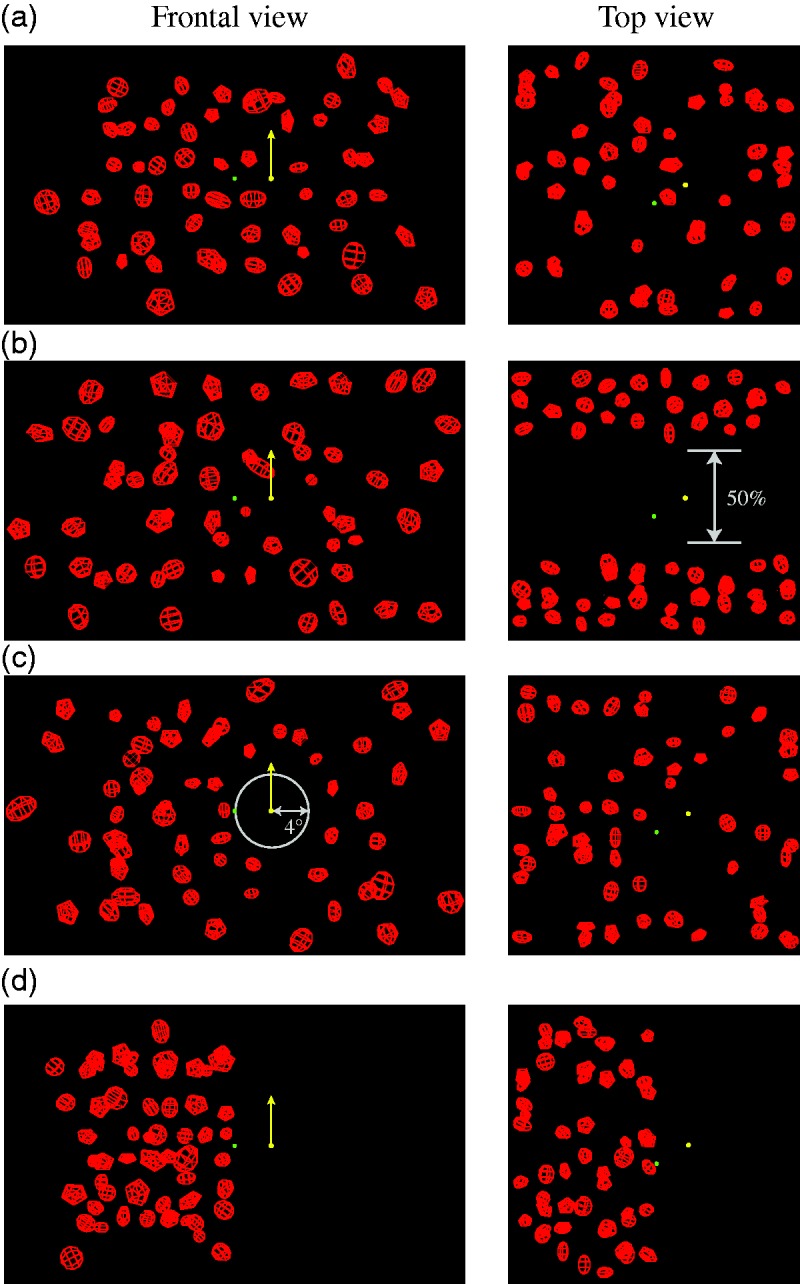
Frontal (left column) and top (right column) views of the displays used in Experiment 1 (red wireframe objects are to scale, the probe and fixation point have been enlarged for clarity). (a) Full display, (b) No local depth, (c) No local frontal, (d) Hemifield. The yellow dot probe moved vertically in the scene (yellow arrow) and the green dot the fixation point which was placed at the screen distance and had zero disparity. Each display is depicted at the time point in which the probe is at the midpoint of its trajectory.

In this experiment, we addressed this question using a retinal motion nulling paradigm (see also Niehorster & Li, 2012; Rushton, Foulkes, & Warren, 2013) to measure the flow parsing gain which indicates the extent to which the visual system can identify and subtract the retinal motion component resulting from self-motion to recover scene-relative object motion. The displays simulated an observer moving through a cloud of wireframe objects. A probe dot at the center depth of this cloud was placed to the left or right of a central fixation point and moved vertically through the cloud. Four display conditions were tested: In the *full* display condition (Figure 1(a)), objects were placed in depth throughout the depth range of the viewing frustum. There were objects close to the probe, both in depth and in the frontal view of the display, providing local motion information around the probe. In the *no local depth* display condition (Figure 1(b)), no objects were placed in the center half of the depth range of the viewing frustum. This removed local motion information that originated from similar depths as the probe object, but kept the local motion information from objects in the retinal vicinity of the probe intact. In the *no local frontal view* display condition (Figure 1(c)), objects were placed in depth throughout the depth range of the viewing frustum, but no objects were placed within 4° of the probe object in the frontal view. This removed local motion information originating from objects in the retinal vicinity of the probe, but left motion information from objects at similar depths as the probe object intact. Last, in the *hemifield* display condition (Figure 1(d)), similar to a display used by Warren and Rushton (2009b), no objects were placed in the hemifield containing the probe. This generated a stronger test of flow parsing as only global motion information was available to estimate and subtract the component of retinal motion due to self-motion.

**Figure 2.**
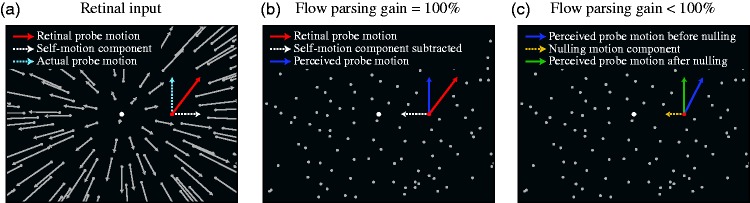
Schematic illustration of flow parsing. Panel (a) depicts the input instantaneous retinal velocity field, (b) the perceived probe motion with complete flow parsing, and (c) the perceived probe motion with incomplete flow parsing. The yellow dotted arrow in (c) indicates the nulling retinal motion component toward the FOE determined by the adaptive staircase procedure such that the perceived probe motion is vertical.

Figure 2 schematically illustrates the instantaneous velocity field in the displays presented in our experiment and illustrates how we measured the flow parsing gain. Figure 2(a) depicts the radial flow field shown to observers in the full display condition. The focus of expansion (FOE) of this radial flow field (white dot) corresponds to the observer’s heading direction. As the probe dot moves through the cloud and is simulated to be approached by the observer, its retinal motion (red arrow) is the vectorial combination of its vertical motion in the scene (cyan dotted arrow) and a motion component away from the FOE resulting from the simulated forward self-motion (white dotted arrow). To accurately recover the scene-relative probe motion, an observer would need to completely remove, or *parse out*, the self-motion component from the probe’s retinal motion. This is equivalent to flow parsing adding a motion component to the probe’s retinal motion that is opposite to the direction of the flow component in the probe’s retinal motion. This component is toward the FOE (white dotted arrow in Figure 2(b)) and cancels out the self-motion component in the probe’s retinal motion such that the probe is perceived to move vertically in the scene.

Figure 2(b) illustrates the perceived scene-relative probe motion when the self-motion component is completely subtracted from the probe’s retinal motion, that is, the compensation gain of flow parsing is 100%. However, when the gain of the flow parsing process is less than 100%, the incomplete removal of the self-motion component leads to the perception of some residual probe motion away from the FOE (Figure 2(c), blue arrow). In our method, we measure the extent to which the flow parsing process removes the self-motion component, as a percentage of the total self-motion component in the retinal motion of the probe. To this end, we null the probe’s residual perceived retinal motion due to self-motion by adding a motion component toward the FOE (yellow dotted arrow) to the probe’s retinal motion under the control of an adaptive staircase procedure (Kontsevich & Tyler, 1999). We can then find the point of subjective equality (PSE) at which the probe is perceived to move vertically in the scene. Our displays are set up such that when probe motion is perceived to be vertical, the complete self-motion component is removed, possibly by a combination of the flow parsing process and the extra nulling component that cancels out residual perceived probe motion away from the FOE. Therefore, because the magnitude of the nulling component required to achieve the perception of vertical probe motion corresponds to the remaining part of the self-motion component that is not removed by flow parsing, the flow parsing gain is computed as: 1−(PSE nulling speed) / (speed of the self-motion component), that is, 1−(length of yellow arrow in Figure 2(c)) / (length of white arrow in Figure 2(b)).

If observers could accurately perceive scene-relative object motion during forward self-motion using visual information alone, we expected no nulling component to be added for the probe to be perceived to move vertically, which corresponds to a unity flow parsing gain. However, flow parsing gains significantly below unity were expected if flow parsing could not accurately recover scene-relative object motion based on visual information alone. Furthermore, if either form of local motion information we examined in this experiment was not used for the perception of scene-relative object motion, we expected the flow parsing gain to remain constant across the four display conditions. In contrast, if local motion information originating from the same depth as the probe object, or in the retinal vicinity of the probe object was used for the perception of scene-relative object motion, this would be evident from lower flow parsing gains for the display conditions where the respective type of local motion information was removed compared with when it was not removed.

### Methods

#### Observers

Twelve students and staff (11 naïve to the specific goals of the study; 8 males, 4 females) between the age of 18 and 31 years at the University of Hong Kong participated in the experiment. All had normal or corrected-to-normal vision and provided informed consent approved by the Human Research Ethics Committee for Non-Clinical Faculties at The University of Hong Kong.

#### Visual stimuli and experimental setup

A stereo display (56°H × 33°V, 120 Hz, focal distance and viewing distance 56.5 cm) simulated forward self-motion at 0.30 m/s toward a cloud of 58 red wireframe objects (diameter: 1.2–2.7 cm). The depth range of the cloud was 0.69 to 1.03 m at the beginning of the 1-s trial and was 0.39 to 0.73 m at the end. For reference, the displays presented optic flow equivalent to what would be experienced during forward self-motion at 1 m/s with objects (diameter: 4–9 cm) in the depth range of 2.32 to 3.47 m at the beginning of the trial. Unique wireframe objects were used, and the display was scaled down to promote fusion of the stimulus. The simulated self-motion direction was at the center of the display indicated by a green fixation dot (0.2° diameter). The fixation point always coincided with the FOE at the direction of self-motion to avoid induced motion of the fixation point. A yellow probe dot was placed at the center depth of the cloud and moved vertically up in the scene. The probe (0.25° diameter) was shown for the last 200 ms of motion. The midpoint of the probe’s retinal motion trajectory during this interval was 4° to the left or right of the fixation point, and the probe speed was chosen such that its vertical instantaneous retinal speed at its midpoint was 2°/s. At this eccentricity, the self-motion component in the retinal motion of the probe at its midpoint was also 2°/s. The 4° probe eccentricity was chosen to compromise between placing the probe not too eccentrically and far enough from the FOE so that the probe’s retinal motion contained a significant self-motion component.

Four display conditions were tested. In the *full* condition (Figure 1(a)), the objects were placed on a jittered 10 × 6 grid in the frontal plane, after which their depth was randomly chosen within the entire cloud’s depth range. In the *no local depth* condition (Figure 1(b)), no objects were placed in the center half of the depth range, such that no objects were placed at a similar depth as the probe. In the *no local frontal view* condition (Figure 1(c)), no objects were placed within 4° from the probe in the frontal view of the display, removing local motion information from the retinal vicinity of the probe. At no point during the course of a trial did the probe object overlap with any of the objects in the display. In the *hemifield* condition (Figure 1(d)), no objects were placed in the display on the side of the probe. To ensure a similar magnitude of global flow for all four display conditions, the number of objects was the same for all displays. The average retinal velocity of the objects differed within 8% between conditions due to the differences in 3D layout of the object cloud.

A horizontal motion component toward the FOE was added to the probe’s retinal motion by a Bayesian adaptive staircase procedure (the Psi method, Kontsevich & Tyler, 1999) to find the PSE at which the probe was perceived to move vertically. To accelerate the measurement of the PSE, in each trial, based on the already collected data, this adaptive staircase predicted which nulling horizontal motion component would provide the most information about the observer’s PSE, and then presented it to the observer. The right angle between the component added by the staircase and the vertical probe motion in the scene maximized the sensitivity of measuring the observer’s flow parsing gain.

The displays were programmed in MATLAB using the Psychophysics Toolbox 3 (Brainard, 1997; Pelli, 1997) and were generated on an nVidia Quadro K2000 graphics card. The displays were presented on an Asus VG278H 27″ LCD monitor (resolution 1920 × 1080 pixels) at 120 Hz (60 Hz per eye). Observers viewed the displays through a pair of LCD shutter glasses (nVidia 3D Vision 2) driven by an infrared emitter built into the monitor while their head was stabilized by a chinrest at the viewing distance of 56.5 cm.

#### Procedure

On each trial, the fixation dot first appeared for 1 s. The first frame then appeared for 2 s to allow observers to fuse the display and notice the position of the yellow probe dot and was followed by 1 s of motion in which the probe was only visible for the last 200 ms. The probe was shown for only 200 ms to prevent a perceivable curved motion trajectory due to the fact that the different motion components in the retinal motion of the probe accelerated differently during approach. Observers were asked to fixate the fixation dot throughout the trial. At the end of the motion, a blank screen appeared and observers were asked to use the left and right mouse buttons to indicate whether they perceived the probe moved obliquely leftward or rightward. We did not measure eye movements in the experiment but assumed that observers followed the instructions and were able to maintain their fixation on the fixation dot throughout the trial as validated by previous studies (e.g., Ehrlich, Beck, Crowell, Freeman, & Banks, 1998; Palmisano & Kim, 2009).

Each observer completed four blocks, with each block containing 80 randomized trials (40 trials for each staircase × 2 probe locations [left or right]) for one of the display conditions. The testing order of the display conditions was counterbalanced between observers. To make sure observers understood the task, they received 3 to 5 training trials at the beginning of each block. No feedback was provided on any trial. An experimental session typically lasted 40 min.

#### Data analysis

We fitted a cumulative Gaussian to the response data to determine the PSE nulling speed *v_n_* at which observers perceived the probe to move vertically. To compute the flow parsing gain, we first computed the magnitude of the self-motion component in the retinal motion of the probe (*v_f_*) at the probe’s eccentricity, which is given as:

(1) $$v_{f}=\frac{T\sin\theta }{D}$$

where *T* is the observer’s translation speed, θ is the probe’s eccentricity (the angular distance between the green fixation point and the yellow probe in Figure 1), and *D* the distance of the probe at the midpoint of its trajectory from the observer (see Grindley, 1942; Rieger, 1983).

Because the PSE nulling component (*v_n_*) corresponds to the remaining self-motion component not removed from the probe’s retinal motion by flow parsing (Figure 2(c)), the flow parsing gain is given as:

(2) $$(1-\frac{v_{n}}{v_{f}})\times 100\%$$

Data were analyzed with repeated-measures analyses of variance (ANOVAs), and Newman-Keuls correction was used for post hoc analyses.

### Results

**Figure 3.**
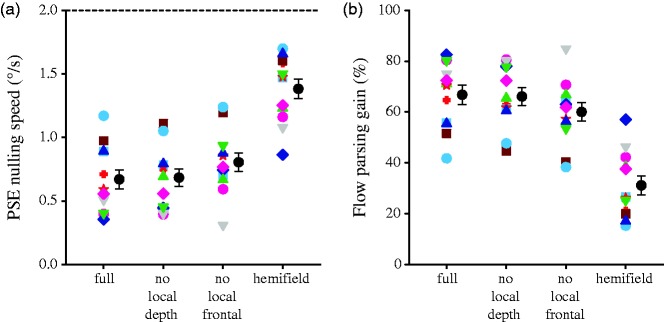
Data of Experiment 1. PSE nulling speed (a) and flow parsing gain (b) for each observer along with the mean for the four display conditions. Error bars are *SE*s across 12 observers, and the dashed line in the upper panels indicates the magnitude of the self-motion component to be subtracted. PSE = point of subjective equality.

Because a 4 (display condition) × 2 (probe location) repeated-measures ANOVA did not reveal any significant effect of probe location (*p* > .614), the flow parsing gain data were averaged over probe location. These flow parsing gains, along with the PSE nulling speeds at which observers perceived the probe to move vertically, are plotted for each display condition in Figure 3. For all displays, the flow parsing gain was significantly higher than 0%, *t*(11) > 8.36, *p* < .0001, and significantly lower than 100%, *t*(11) < –8.91, *p* < .0001. This indicates that while flow parsing relying on visual information alone removed part of the self-motion component, it did not completely remove the self-motion component from the probe’s retinal motion to recover accurate scene-relative object motion.

A one-way repeated-measures ANOVA revealed that the mean flow parsing gains differed significantly across displays, *F*(3, 33) = 89.0, *p* < <.0001. Newman-Keuls post hoc tests revealed that while the mean flow parsing gain was not different for the full (mean gain ± *SE*: 66.8 ± 3.7%) and no local depth display conditions (66.1 ± 3.5%, *p* = .78), it was significantly lower in the no local frontal view condition (60.0 ± 3.6%) than in the full condition (*p* = .030). It was also significantly lower in the hemifield condition (31.3 ± 3.7%) than in the no local frontal view condition (*p* = .0002). This suggests that local motion information in the retinal vicinity of the probe object plays a significant role in flow parsing.

### Discussion

The flow parsing gain data show that flow parsing occurred for all display conditions as consistent with all previous work on this phenomenon (e.g., Warren & Rushton, 2009b). Nevertheless, the flow parsing gains were significantly below 100% (mean < 67%). This indicates that rich visual information about self-motion and the layout of the scene is not sufficient to enable the precise perception of scene-relative object motion during forward self-motion.

As the only difference between the display conditions was the scene layout, differences in flow parsing gains between the displays can be directly related to the type of motion information that was removed. The findings are consistent with previous findings reported in the literature. First, we found that the removal of motion information originating from similar depths as the probe object while keeping local motion information in the retinal vicinity of the probe object did not affect flow parsing. This is consistent with the findings of Warren and Rushton (2007) who reported unchanged perceived tilts of a probe object during simulated lateral or rotational self-motion when the probe object was repositioned outside the depth range of the scene. Second, we observed that flow parsing gains progressively decrease as more local motion in the retinal vicinity of the probe is removed. This indicates that the pattern of local motion near the probe on the image plane plays an important role in perceiving object motion during self-motion. This finding is consistent with the findings of Warren and Rushton (2009b) who reported that the amount of tilt induced in the perceived trajectory of a moving probe object reduced as background flow in the vicinity of the probe was removed. Last, we observed that flow parsing still occurred when the background flow was removed from the hemifield that contained the probe. This is also consistent with the findings reported by Warren and Rushton (2009b).

Different from previous findings, the results from this experiment for the first time quantitatively show how the accuracy of the perception of scene-relative object motion changes when local motion information was removed from the display while global flow information was kept as similar as possible. Specifically, while removing motion from similar depths as the moving object did not cause a significant change in flow parsing gain, removing motion information from the retinal vicinity of the moving object led to a significant decrease in flow parsing gain of 6.7 ± 3.1 percentage points, and removing retinal motion from the entire hemifield containing the probe caused a significant decrease in flow parsing gain of 35.5 ± 2.5 percentage points compared with the full display. All together, these findings show that the perception of object motion during forward self-motion is driven both by global optic flow and local motion processes. The finding that the flow parsing gain in the hemifield display was about half that in the full display indicates that local and global motion processes contribute approximately equally to the perception of scene-relative object motion.

## Experiment 2: Tuning to Self-Motion Speed

To further characterize flow parsing, in this experiment, we examined the tuning of flow parsing gain to self-motion speed using full and hemifield displays similar to those in the previous experiment. Specifically, we varied the simulated forward self-motion speed to change the self-motion component in the probe’s retinal motion (see the white dotted arrow in Figure 2(a)). The speed of the self-motion component was varied between slow (1.6°/s) to fast motion (4.6°/s) in four equal steps. These speeds span a range of commonly experienced self-motion speeds in daily life. Specifically, whereas the slow speed of 1.6°/s can be associated with an observer approaching a cloud of randomly positioned objects (depth range: 2.25–3.5 m) at 1 m/s, the fast speed of 4.6°/s can be associated with an observer approach speed of 2.88 m/s.

Because faster self-motion speeds lead to higher optic flow speeds that can enable more accurate perception and control of self-motion as indicated by lower variability of steering and heading perception responses (Chen, Niehorster, & Li, 2013), and flow parsing utilizes the percept of self-motion, we expect flow parsing gain to increase with the increase of the self-motion component in the probe’s retinal motion. Alternatively, if flow parsing subtracts background motion with a constant gain over a range of self-motion speeds, we would expect the flow parsing gain to remain constant as the speed of the self-motion component in the probe’s retinal motion increases.

### Methods

#### Observers

Eight staff and students (five males and three females; seven naïve to the purpose of the experiment; two also participated in Experiment 1) between the age of 19 and 30 years at the University of Hong Kong participated in the experiment. All had normal or corrected-to-normal vision and provided informed consent approved by the Human Research Ethics Committee for Non-Clinical Faculties at The University of Hong Kong.

#### Visual stimuli and procedure

The full and hemifield displays from Experiment 1 were tested. To examine the tuning of flow parsing to the speed of the self-motion component, we simulated forward observer translation through the cloud of red wireframe objects at 0.24 m/s, 0.39 m/s, 0.54 m/s, or 0.69 m/s. To keep the depth and position of the probe, as well as the depth range of the scene objects and thus the presented range of binocular disparities, constant at the midpoint of the probe’s trajectory for the four translation speeds, the depth range of the cloud at the beginning of the trial was changed as follows 0.54 to 0.84 m, 0.60 to 0.90 m, 0.66 to 0.96 m, and 0.73 to 1.03 m, respectively, for the four translation speeds. These translation speeds corresponded to self-motion component speeds of 1.6°/s, 2.6°/s, 3.6°/s, and 4.6°/s, respectively. The probe’s vertical retinal speed at the midpoint of its trajectory remained unchanged at 2°/s, and the displays were otherwise identical to those in Experiment 1. For reference, the displays presented optic flow equivalent to what would be experienced during forward self-motion at 1 m/s, 1.63 m/s, 2.25 m/s, or 2.88 m/s with objects in the depth range of 2.25 to 3.5 m, 2.5 to 3.75 m, 2.75 to 4.0 m, or 3.04 to 4.29 m, respectively.

The procedure for each trial was the same as in Experiment 1, except that the presentation time of the motion was shortened to 500 ms for all translation speeds. This was to accommodate the larger translation speeds. Each observer completed two blocks, with each block containing 360 randomized trials (40 trials for each staircase × 2 probe locations [left or right] × 4 self-motion speeds) for one of the display conditions. The testing order of the display conditions was counterbalanced between observers. An experimental session typically lasted 40 min.

### Results

**Figure 4.**
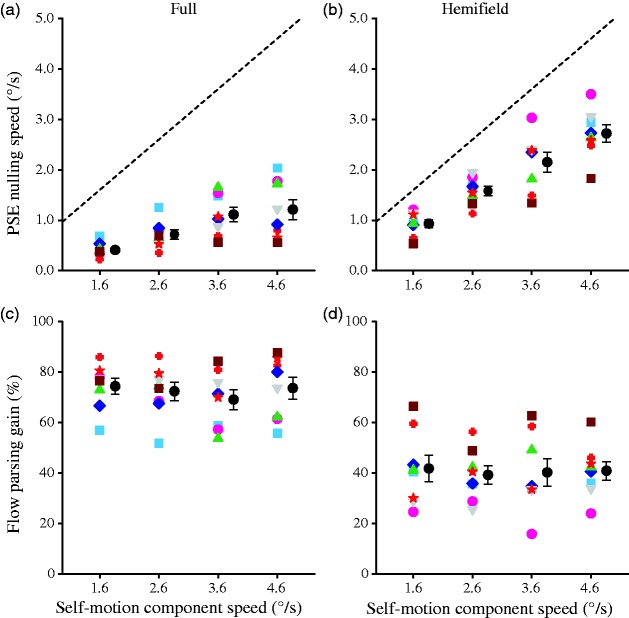
Data of Experiment 2. PSE nulling speed (top row) and flow parsing gain (bottom row) against self-motion component speed for each observer along with the mean for the full (left column) and the hemifield (right column) display conditions. Error bars are *SE*s across eight observers, and the dashed line in the upper panels indicates the magnitude of the self-motion component to be subtracted. PSE = point of subjective equality.

A 2 (display condition) × 4 (self-motion speed) × 2 (probe location) repeated-measures ANOVA did not reveal any significant effect of probe location (*p* > .081), the flow parsing gain data were thus averaged over probe location. These flow parsing gains, along with the PSE nulling speeds at which observers perceived the probe to move vertically, are plotted against self-motion speed for each display condition in Figure 4. In both display conditions and for all self-motion speeds, the flow parsing gain was significantly smaller than 100%, *t*(7) < −6.02, *p = *.0001, indicating that flow parsing did not completely remove the self-motion component from the probe’s retinal motion.

A 2 (display condition) × 4 (self-motion speed) repeated-measures ANOVA revealed that while the main effect of display condition was significant, *F*(1, 7) = 71.3, *p* < .0001, the main effect of self-motion speed and their interaction were not significant, *F*(3, 21) = 0.95, *p* = .44 and *F*(3, 21) = 0.55, *p* = .65, respectively. The consistently lower flow parsing gain in the hemifield than in the full display condition replicated the findings from Experiment 1.

### Discussion

Because faster self-motion speeds lead to higher optic flow speeds that can allow for more accurate perception and control of self-motion (Chen et al., 2013), we expected the gain of flow parsing, which likely utilizes the percept of self-motion, to increase with increasing self-motion speed. Contrary to this expectation, for both the full and hemifield displays, the flow parsing gain did not change when forward self-motion speed increased, indicating that the flow parsing gain is not tuned to self-motion speed. Instead, flow parsing was observed to subtract a constant proportion of background flow from the retinal motion of the probe object. This indicates that the retinal motion component removed by flow parsing is perfectly tuned to self-motion speed.

## Experiment 3: Tuning to Object Motion Speed

In this experiment, we examined the tuning of flow parsing gain to object motion speed using the full and hemifield displays. Specifically, we varied the probe’s moving speed through the cloud to change the object motion component in the probe’s retinal motion (see the cyan dotted arrow in Figure 2(a)). The speed of the object motion was varied between slow (1.6°/s) to fast motion (4.6°/s) in four equal steps.

Because the increase of object motion speed has no effect on the background flow, we expect the gain of flow parsing in subtracting the background flow from the object retinal motion to remain constant despite the increase of the object motion speed. An alternative prediction is that the flow parsing gain will increase with the increase of object motion speed due to the fact that the higher the speed of the moving object, the more it stands out from the background optic flow (Royden & Moore, 2012), thus making the perception of the object motion easier and thereby the flow parsing gain higher. Specifically, given a constant self-motion speed, the retinal motion of a fast moving object has a larger signal-to-noise ratio of object motion component to self-motion component than does a slow moving object, which could make it easier to parse out the self-motion component and recover scene-relative object motion.

### Methods

#### Observers

Eight students and staff (all naive to the purpose of the experiment; six males, two females; none participated in the previous two experiments) between the age of 19 and 26 years at the University of Hong Kong participated in the experiment. All had normal or corrected-to-normal vision and provided informed consent approved by the Human Research Ethics Committee for Non-Clinical Faculties at The University of Hong Kong.

#### Visual stimuli and procedure

The full and hemifield displays from Experiment 1 were tested. To examine the tuning of flow parsing to object motion speed, the probe moved through the cloud at 1.6°/s, 2.6°/s, 3.6°/s, and 4.6°/s, respectively. The depth range of the cloud at the start of the trial was 0.57 to 0.87 m, and the simulated forward self-motion speed was 0.30 m/s, such that the probe was at the same position in the middle of its trajectory as in Experiment 2, and the self-motion component in the retinal motion of the probe at its midpoint was kept at 2°/s. The displays were otherwise identical to those in Experiment 1.

The procedure for each trial was the same as in Experiment 2. The presentation time of the motion for all object speeds was 500 ms. Each observer completed two blocks, with each block containing 360 randomized trials (40 trials for each staircase × 2 probe locations [left or right] × 4 object motion speeds) for one of the display conditions. The testing order of the display conditions was counterbalanced between observers. An experimental session typically lasted 40 min.

### Results

**Figure 5.**
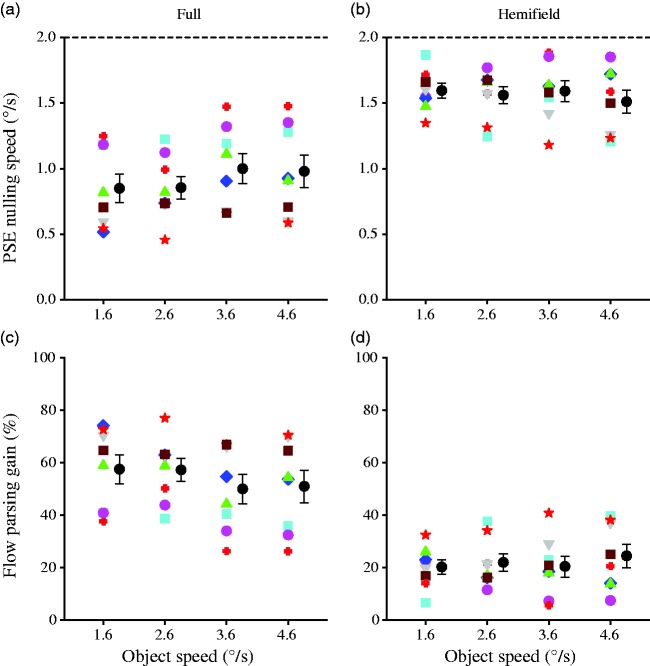
Data of Experiment 3. PSE nulling speed (top row) and flow parsing gain (bottom row) against object speed for each observer along with the mean for the full (left column) and the hemifield (right column) display conditions. Error bars are *SE*s across eight observers, and the dashed line in the upper panels indicates the magnitude of the self-motion component to be subtracted. PSE = point of subjective equality.

Because a 2 (display condition) × 4 (object motion speed) × 2 (probe location) repeated-measures ANOVA did not reveal any significant effect of probe location (*p* > .11), the flow parsing gain data were averaged over probe location. The flow parsing gains, along with the PSE nulling speeds at which observers perceived the probe to move vertically, are plotted against object motion speed for each display condition in Figure 5. In both display conditions and at all object motion speeds, the flow parsing gain was significantly smaller than 100%, *t*(7) < −7.75, *p < *.0001, indicating that flow parsing did not completely remove the self-motion component from the probe’s retinal motion.

A 2 (display condition) × 4 (object motion speed) repeated-measures ANOVA revealed that while the main effect of display condition was significant, *F*(1, 7) = 59.1, *p* = .0001, the main effect of object motion speed was not, *F*(3, 21) = 0.99, *p* = .42, and their interaction effect was marginally significant, *F*(3, 21) = 3.04, *p* = .051. The consistently lower flow parsing gain in the hemifield than in the full display condition replicates the findings from Experiment 1. A Newman-Keuls post hoc test showed that the marginally significant interaction was caused by a small drop in flow parsing gain for the full display when the object speed increases from 2.6°/s to 3.6°/s (*p* = .047).

### Discussion

The data show that flow parsing gain for both display conditions is not tuned to object motion speed. This indicates that despite the fact that faster moving objects stand out more from the background optic flow (Royden & Moore, 2012), flow parsing was not enhanced by increasing object motion speed. Instead, flow parsing gain remained constant as object speed increased.

## General Discussion

In this study, we investigated how accurately observers can perceive scene-relative object motion during forward self-motion using visual information alone. We developed and employed a retinal motion nulling paradigm to directly measure flow parsing gain to quantify the accuracy with which the visual system subtracts the self-motion component from the object’s retinal motion for the recovery of scene-relative object motion during self-motion. Our results help develop a more detailed understanding of the role local motion information plays in flow parsing, as well as how flow parsing is tuned to self-motion and object motion speed. Our results inform computational and neural models of flow parsing (e.g., Layton & Fajen, 2016) and provide the data required for validating these models.

Most previous studies only qualitatively examined flow parsing (e.g., Rushton & Warren, 2005; Warren & Rushton, 2008, 2009a, 2009b). For the three studies that have quantitatively measured flow parsing gain, Dupin and Wexler (2013) examined the perception of the rotation of a plane during lateral self-translation. It is unclear how their findings can be related to the common case of perceiving object motion during forward self-motion. Warren and Rushton (2007) fitted a linear model to the perceived tilt of the probe’s motion trajectory due to flow parsing. Dokka, MacNeilage, DeAngelis, and Angelaki (2015) assessed flow parsing gain by also measuring the extent to which flow parsing tilted the perceived motion direction of a probe. Using the perceived tilt of the probe’s motion trajectory for the estimation of flow parsing as in these two studies is indirect and susceptible to bias compared with the nulling procedure we used in the current study that directly measured the magnitude of the subtracted self-motion component during flow parsing. Furthermore, observers in the study by Warren and Rushton (2007) reproduced the amount of motion trajectory tilt with a dial at the end of the trial based on their remembered probe motion trajectory. Our 2AFC retinal motion nulling paradigm did not involve such memory or reproduction components and is thus relatively impenetrable to cognitive influences (see Gogel, 1990). Last, these three studies all examined flow parsing during sideways self-motion. In contrast, in this article, we quantitatively measured the accuracy of flow parsing during forward self-motion, a commonly experienced type of self-motion in daily life.

Combining the findings from the three experiments in the current study, we derive the following conclusions. First, the results of Experiment 1 revealed that removing local motion information originating from objects at a similar depth as the probe object did not affect flow parsing accuracy. This is consistent with the findings of Warren and Rushton (2007) and indicates that the visual system is able to reconstruct the speed of the self-motion component at the probe location even when it is not directly represented in the optic flow. It should be noted that adequate information about the depth of the probe in the scene is probably important for this (see Gogel, 1990), as is also suggested by the finding of Warren and Rushton (2009a) that observers made fewer errors in judging the direction of scene-relative probe motion as more depth cues were added to the display.

Second, the results of Experiment 1 also revealed that local motion information stemming from scene objects in the retinal vicinity of the moving object is important for object motion perception during self-motion. This is supported by a decrease in flow parsing gains when scene objects within 4° from the probe object were removed and a further decrease to half the gain of the full display when all objects in the hemifield at the side of the probe were removed. Given that the global flow magnitude was equated in the full and the hemifield displays, the finding that the flow parsing gain in the hemifield display is about half of that in the full display suggests that local and global motion processes contribute approximately equally to the perception of scene-relative object motion. This is similar to the finding of Warren and Rushton (2009b), who by removing all objects in the hemifield of the probe found that the magnitude of the flow parsing effect decreased by about 40%. The small difference (<10%) in the contribution of local motion to flow parsing found between their study and ours could be due to many differences between their and our displays, such as the type of objects used (we used wireframe objects and they used dots) and the absence of depth information in their display.

Third, the flow parsing gain data from Experiments 2 and 3 show that the flow parsing gain is not tuned to self-motion or object motion speed, at least in the range that we tested in the current study. Specifically, when the speed of self-motion or object motion was varied, the flow parsing gain remained constant. As such, the retinal motion component removed by flow parsing was perfectly tuned to self-motion speed. The absence of tuning of the gain of flow parsing, a global subtraction process, resembles the known absence of tuning of the local motion contrast phenomenon, that also subtracts a constant proportion of the background motion over a large range of object and background motion speeds (Farrell-Whelan et al., 2012; Gogel, 1979; Post et al., 1989).

Last, our results show that flow parsing gains remain significantly smaller than unity (<75% across all experiments) even though all displays contained sufficient depth information to specify the object’s 3D position in the scene. This finding is consistent with the results of our previous study (Niehorster & Li, 2012) that tested flow parsing of a probe dot moving over a large ground plane (83°H × 83°V), which yielded a mean flow parsing gain of 69% (SE: ±3%). These results together indicate that the perception of scene-relative object motion during self-motion using visual information alone is not accurate. Our results underscore the claim that when available, the brain also uses nonvisual information about self-motion, such as vestibular and proprioceptive information and efference copies of motor commands generated during body and limb movements, to enable the accurate perception of scene-relative object motion during self-motion in our daily life (Gogel, 1990; Wallach, 1987; Warren & Rushton, 2007). Recent research findings have shown that such nonvisual information is indeed used to detect and perceive scene-relative object motion during self-motion (e.g., Dokka et al., 2015; Dupin & Wexler, 2013; Fajen & Matthis, 2011, 2013; MacNeilage, Zhang, DeAngelis, & Angelaki, 2012; Tcheang, Gilson, & Glennerster, 2005; Van Pelt & Medendorp, 2007).
